# Infection-Induced SARS-CoV-2 Seroprevalence among Blood Donors, Japan, 2022

**DOI:** 10.3201/eid2909.230365

**Published:** 2023-09

**Authors:** Ryo Kinoshita, Takeshi Arashiro, Noriko Kitamura, Satoru Arai, Koki Takahashi, Tadaki Suzuki, Motoi Suzuki, Daisuke Yoneoka

**Affiliations:** National Institute of Infectious Diseases, Tokyo, Japan (R. Kinoshita, T. Arashiro, N. Kitamura, S. Arai, T. Suzuki, M. Suzuki, D. Yoneoka);; Japanese Red Cross Society, Tokyo, Japan (K. Takahashi)

**Keywords:** SARS-CoV-2, COVID-19, respiratory infections, viruses, zoonoses, seroprevalence, surveillance, seroepidemiology, Japan

## Abstract

A nationwide survey of SARS-CoV-2 antinucleocapsid seroprevalence among blood donors in Japan revealed that, as of November 2022, infection-induced seroprevalence of the population was 28.6% (95% CI 27.6%–29.6%). Seroprevalence studies might complement routine surveillance and ongoing monitoring efforts to provide a more complete real-time picture of COVID-19 burden.

SARS-CoV-2 transmission has been identified in Japan since early 2020, and by the end of 2022, ≈29 million COVID-19 diagnosed cases had been reported (*1*). However, case-based surveillance of COVID-19 may underestimate the total number of infections because undiagnosed persons with mild symptoms or asymptomatic infection might not seek treatment. Seroprevalence studies targeting the SARS-CoV-2 nucleocapsid antibody can be used to estimate the proportion of persons experiencing natural infection and may provide insights to understand population immunity status independent of vaccination (*2*–*5*).

During June 2020–February 2022, four large-scale serologic surveys were conducted in 5 prefectures of Japan (Miyagi, Tokyo, Aichi, Osaka, and Fukuoka) (*6*,*7*); a comprehensive survey covering all 47 prefectures has not yet been conducted. Seroprevalence during SARS-CoV-2 Omicron variant predominance in February 2022 was 3.5% for the 5 prefectures (*6*). However, after the seventh epidemic wave started in July 2022, we analyzed residual blood donation samples to determine infection-induced seroprevalence levels in all 47 prefectures of Japan to monitor trends in community transmission across the nation (Figure 1, panel A). This study was performed as an active epidemiologic investigation in accordance with the Act on the Prevention of Infectious Diseases and Medical Care for Patients with Infectious Diseases (Infectious Disease Control Law of Japan; Act No. 114 of 1998) and did not require formal ethics review or participant consent. 

**Figure 1:**
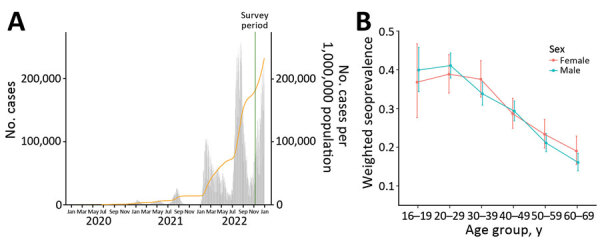
Reported COVID-19 cases among the general population and seroprevalence of SARS-CoV-2 among blood donors, Japan. A) Number of reported COVID-19 cases by date of report, 2020–2022. Orange line indicates cumulative number of reported cases per 1 million persons. Green shading indicates survey period. B) Weighted seroprevalence stratified of SARS-CoV-2 among blood donors from November 6–13, 2022, by age group and sex. Error bars represent 95% CIs.

## The Study

Participants were blood donors to the Japanese Red Cross Society during November 6–13, 2022 (Figure 1, panel A). To be included, participants had to be 16–69 years of age at the time of donation and have provided whole blood or blood components. Persons were not permitted to donate blood if they had been diagnosed with or tested positive for COVID-19 and were <4 weeks after symptom resolution or, for asymptomatic persons, sample collection; if they had acute COVID-19–related signs or symptoms (e.g., fever, cough, breathing difficulty) or experienced altered senses of taste or smell during the period between 2 weeks after symptom onset and 3 days after resolution; or if they were close contacts of confirmed COVID-19 case-patients and <2 weeks after most recent contact. 

We calculated the necessary number of samples on the basis of prefecture-level population sizes in October 2021 and expected COVID-19 prevalence from the cumulative number of cases as of September 1, 2022. We also extracted data on age and sex. We tested serum from randomly selected blood samples collected from eligible blood donors using Elecsys Anti-SARS-CoV-2 (Roche Diagnostics, https://www.roche.com), using the manufacturer-recommended seropositivity cutoff index of ≥1.0. We based seroprevalence estimates on weighted tabulation (*8*). We stratified seroepidemiologic data by prefecture, sex, and age group (16–19, 20–29, 30–39, 40–49, 50–59, or 60–69 years of age) to estimate the survey weights to adjust for the age and sex distribution of each prefecture. We used estimated population as of October 1, 2021, as baseline. We calculated 95% CIs by using the binomial exact method and set the statistical significance level at <0.05 with an acceptable 5% margin of error. For comparison, we also extracted the cumulative number of reported COVID-19 cases through October 30, 2022, from the Ministry of Health, Labour and Welfare (*1*). We extracted vaccination coverage records as of October 30, 2022, from the Digital Agency Vaccination Record System (*9*). 

**Figure 2:**
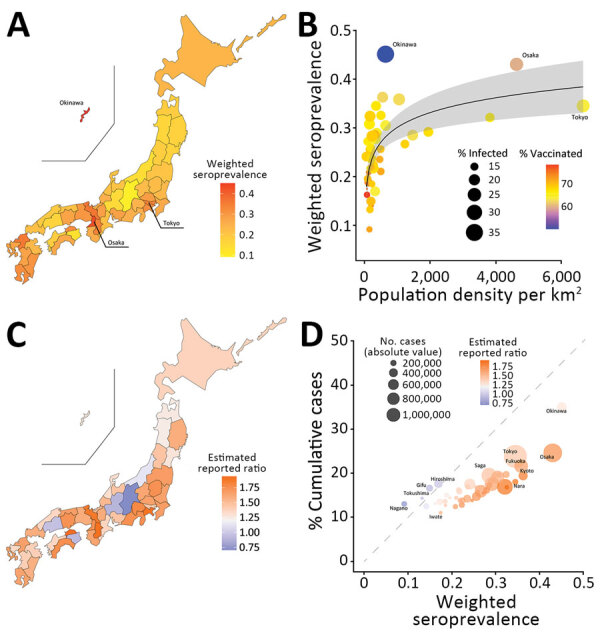
Analyses of weighted seroprevalence of SARS-CoV-2 among blood donors from November 6–13, 2022, compared with reported cases among the general population, Japan. A) Weighted seroprevalence by prefecture. B) Relationship between population density (persons/km^2^) and weighted seroprevalence by prefecture. Circle sizes indicate percentage of infected persons based on cumulative number of reported cases; colors indicate vaccination coverage for >3 doses of COVID-19 vaccine. C) Ratio of weighted seroprevalence to the percentage of cumulative number of reported cases (estimated to reported infections) by prefecture. D) Relationship between weighted seroprevalence and percentage of cumulative number of reported cases by prefecture. Circle sizes represent absolute value of cumulative number of reported cases.

We tested 8,260 specimens from the November 6–13, 2022, study period. Infection-induced seroprevalence in the total population of Japan was 28.6% (95% CI 27.6%–29.6%). We stratified seroprevalence by age and sex (Figure 1, panel B). Median age of blood donors was 47 years (interquartile range 35–55 years). Among both sexes, prevalence peaked in the 20–29-year age group, in which 41.1% (95% CI 37.9%–44.4%) of men and 38.9% (95% CI 34.0%–43.9%) of women were seropositive. Prevalence decreased with age and was lowest among the 60–69-year age group: 16.1% (95% CI 13.9%–18.4%) of men and 19.0% (95% CI 15.6%–22.9%) of women were seropositive. The populations of Tokyo (34.5%, 95% CI 28.7%–40.7%), Osaka (43.0%, 95% CI 36.9%–49.3%), and Okinawa (45.1%, 95% CI 39.7%–50.6%) prefectures had higher seroprevalence than we found overall (Figure 2, panel A) (*10*). 

Except in Okinawa, population density and COVID-19 prevalence appeared to have an exponential relationship (Figure 2, panel B), similar to an observed trend in the United States (*11*). Percentages of reported cases among total population were higher than percentages of seroprevalent nucleocapsid antibodies in several low–population density prefectures: 13.0% versus 9.2% in Nagano, 16.6% versus 14.9% in Gifu, 17.6% versus 17.0% in Hiroshima, and 14.3% versus 13.2% in Tokushima (Figure 2, panels C, D). In most prefectures, however, percentage prevalence of nucleocapsid antibodies was higher than percentage of reported cases among the population, although 95% CIs overlapped. 

## Conclusions

Using specimens from blood donations accepted in November 2022, we revealed the prevalence of nucleocapsid antibodies to SARS-CoV-2 in Japan, although we observed differences in prevalence among prefectures. For comparison over time, a previous population-based serial cross-sectional seroepidemiologic survey showed that prevalences were 3.1% in Tokyo, 4.1% in Osaka, and 1.9% in Fukuoka in December 2021 and 6.4% in Tokyo, 6.1% in Osaka, and 3.3% in Fukuoka in February 2022 (*6*). 

Even after the country was largely affected by Omicron-variant disease, estimated seroprevalence was remarkably lower in Japan (28.6%) than what has been reported in the United Kingdom using blood donor samples taken during October 26–December 16, 2022; antinucleocapsid seroprevalence in the United Kingdom was 82.5% (*12*). Seroprevalence in Japan in November 2022 was comparable to the estimated seroprevalence of 28.8% among blood donors in the United States as of December 2021 (*13*). Lower seroprevalence in Japan might reflect high vaccination coverage or adherence to public health and social measures. Both the United States and the United Kingdom observed similar decreasing prevalence among older age groups (*12*–*14*). Case ascertainment rate was higher in Japan than the United States, where reported infection-induced seroprevalence was 2.2–3.1 times higher than the cumulative number of reported cases (*14*). 

Among limitations in this study, the first is that we adjusted demographic differences among prefectures by survey weights, but selection bias caused by the characteristics of blood donors remains. Second, eligible age in Japan for blood donation is 16–69 years of age; therefore, we could not use these data to evaluate children <16 or elderly persons >69 years of age. Third, samples were all collected within a single 1-week timeframe, which hindered exploration of temporal trends. Finally, ratio of estimated to reported infections does not consider the sensitivity or decay of nucleocapsid antibodies, which could potentially reduce detection of previously infected persons, depending on time since infection, age, sex, and vaccination status (*15*). 

Despite those limitations, we comprehensively evaluated the proportion of infected persons in the overall population of Japan. Although reporting all COVID-19 cases has become increasingly challenging in most countries, seroprevalence studies could potentially complement routine surveillance and continued monitoring over time to provide a more complete real-time picture of COVID-19 burden. 
